# Magnesium homeostasis protects *Salmonella* against nitrooxidative stress

**DOI:** 10.1038/s41598-017-15445-y

**Published:** 2017-11-08

**Authors:** Travis J. Bourret, Lin Liu, Jeff A. Shaw, Maroof Husain, Andrés Vázquez-Torres

**Affiliations:** 1 Department of Medical Microbiology and Immunology, 2500 California Plaza, Creighton University, Criss I, Rm 521, Omaha, NE 68178 USA; 2 Department of Immunology and Microbiology, University of Colorado School of Medicine, Aurora, CO 80045 USA; 3 Veterans Affairs Eastern Colorado Health Care System, 1055 Clermont Street, Denver, DO 80220 USA; 4 Present Address: Division of Pulmonary, Allergy, and Critical Care Medicine, Department of Medicine, University of Alabama Birmingham, Birmingham, Alabama 35294 USA

## Abstract

The PhoPQ two-component regulatory system coordinates the response of *Salmonella enterica* serovar Typhimurium to diverse environmental challenges encountered during infection of hosts, including changes in Mg^2+^ concentrations, pH, and antimicrobial peptides. Moreover, PhoPQ-dependent regulation of gene expression promotes intracellular survival of *Salmonella* in macrophages, and contributes to the resistance of this pathogen to reactive nitrogen species (RNS) generated from the nitric oxide produced by the inducible nitric oxide (NO) synthase of macrophages. We report here that *Salmonella* strains with mutations of *phoPQ* are hypersensitive to killing by RNS generated *in vitro*. The increased susceptibility of ∆*phoQ Salmonella* to RNS requires molecular O_2_ and coincides with the nitrotyrosine formation, the oxidation of [4Fe-4S] clusters of dehydratases, and DNA damage. Mutations of respiratory NADH dehydrogenases prevent nitrotyrosine formation and abrogate the cytotoxicity of RNS against ∆*phoQ Salmonella*, presumably by limiting the formation of peroxynitrite (ONOO^−^) arising from the diffusion-limited reaction of exogenous NO and endogenous superoxide (O_2_^•−^) produced in the electron transport chain. The mechanism underlying PhoPQ-mediated resistance to RNS is linked to the coordination of Mg^2+^ homeostasis through the PhoPQ-regulated MgtA transporter. Collectively, our investigations are consistent with a model in which PhoPQ-dependent Mg^2+^ homeostasis protects *Salmonella* against nitrooxidative stress.

## Introduction

Mutations in *phoPQ* attenuate *Salmonella* virulence by at least 10,000-fold^1–3^. The attenuated phenotype of *phoPQ* mutants has been associated with poor intracellular survival in macrophages, defective activation of *Salmonella* pathogenicity island 2 (SPI2) transcription, and hypersensitivity to defensins, antimicrobial peptides, divalent cations, iron, acid and bile salts^1,4–10^. PhoPQ signaling also boosts antioxidant defenses through the positive regulation of the *sodCI-*encoded superoxide dismutase, the posttranslational stabilization of the alternative σ^S^ factor, and the limitation in the availability of free iron^7,11,12^. In addition, PhoPQ lessens the cytotoxicity of reactive nitrogen species (RNS) generated by inducible nitric oxide synthase (iNOS) in the innate response of mononuclear phagocytic cells^13^.

The antimicrobial activity of NO is best demonstrated in IFNγ-activated phagocytes; however, very little anti-*Salmonella* activity is derived from iNOS expressed through the innate recognition of *Salmonella* lipopolysaccharide by host-cell Toll-like receptor 4^14–19^. There are several possible explanations underlying the marked resistance of *Salmonella* to the nitrosative species synthesized by iNOS during the innate response of professional phagocytes. The low NO fluxes generated in the innate response dramatically limit the synthesis of autooxidative products such as dinitrogen trioxide (N_2_O_3_), which has been associated with sustained anti-*Salmonella* activity of IFNγ-primed macrophages^17^. On the other hand, the SPI2 type III secretion system, the Hmp flavohemoprotein, and low-molecular weight thiols protect *Salmonella* against moderate NO rates generated in the innate immune response^20–22^. As just mentioned, we have recently shown that PhoPQ signaling enhances the intracellular fitness of *Salmonella* by antagonizing the innate host response associated with NO^13^. The mechanism by which the PhoPQ two-component regulatory system defends *Salmonella* against the antimicrobial actions of NO congeners remains unknown. The investigations presented herein have revealed that the PhoPQ two-component regulatory system enhances the resistance of *Salmonella* against the nitrooxidative stress generated in the interaction of exogenous NO with endogenously produced O_2_^•−^ through its regulation of intracellular Mg^2+^ concentrations.

## Results

### PhoPQ-deficient *Salmonella* are hypersusceptible to NO

**Figure 1:**
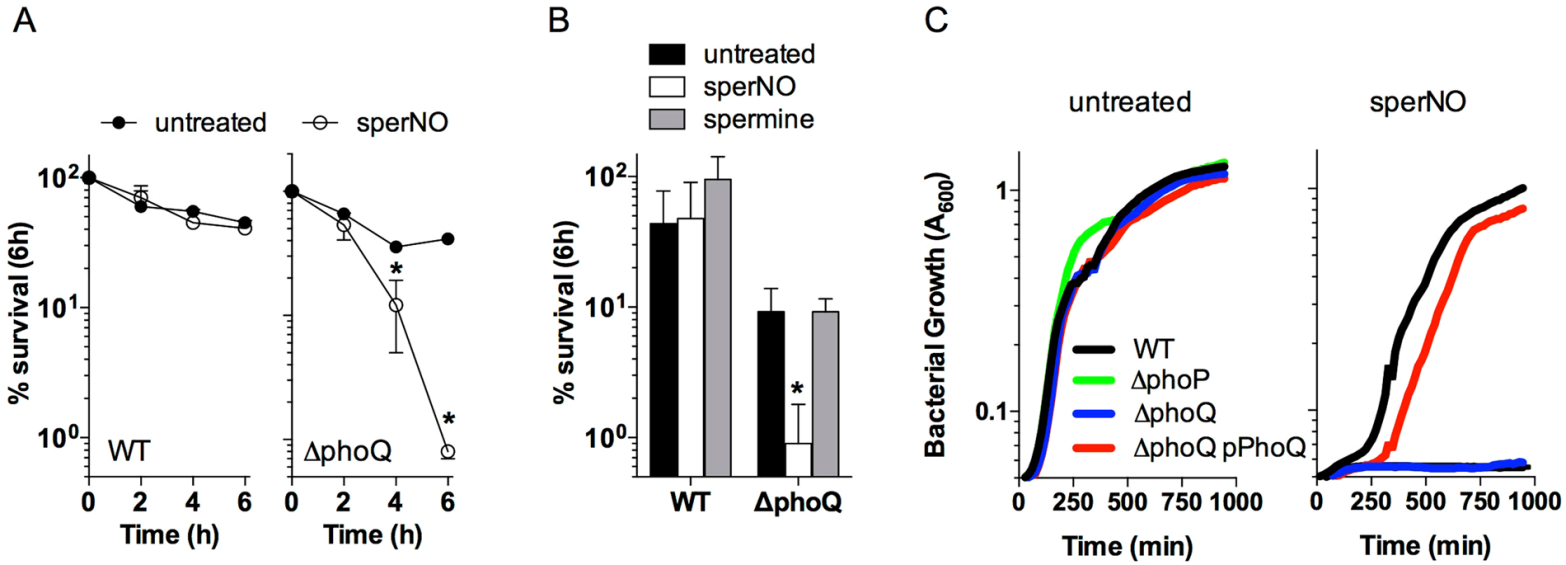
The PhoPQ two-component regulatory system protects *Salmonella* against RNS cytotoxicity. The susceptibility of wild-type (WT) and ∆*phoQ Salmonella* to killing by 250 µM spermine NONOate (sperNO) in PBS at 37 °C was compared after 0, 2, 4 and 6 h (**A**). The bactericidal capacity of 250 µM sperNO was compared to the polyamine base spermine following 6 h of incubation at 37 °C (**B**). Results represent the mean % survival ± SD of 4 independent observations collected from two separate experiments. *P < 0.01 compared to untreated controls. To determine effects of RNS on the growth of *Salmonella*, strains were cultured in LB broth in the presence or absence of 2.5 mM sperNO at 37 °C. Growth was determined by measuring the optical density (OD_600nm_) over time in 96-well microtiter plates using a Biotek Cytation 5 multi-mode plate reader (**C**). Data represent the mean optical density of 3 biological replicates.

The PhoPQ signaling cascade coordinates important aspects of the antioxidant and antinitrosative defenses of *Salmonella* ^12,13^. The PhoPQ two component regulatory system is involved in *Salmonella* defense against Fenton-mediated oxidative stress^7^, however, it is unclear how PhoPQ signaling promotes resistance to RNS. To learn more about the role of PhoPQ in resistance of *Salmonella* to RNS, we investigated the survival of a ∆*phoQ* mutant exposed to the NO generator spermine NONOate (sperNO). Most wild-type *Salmonella* survived 6 h after challenge with 250 µM sperNO, while ∼99% of ∆*phoQ Salmonella* were killed upon sperNO treatment (Fig. 1A). The NO-mediated killing of ∆*phoQ Salmonella* was already noted after 4 h of challenge. The susceptibility of ∆*phoQ Salmonella* to sperNO appears to rely on the generation of NO, because the polyamine spermine control lacked antimicrobial activity (Fig. 1B). A dose-dependent inhibition of growth by sperNO was observed for both ∆*phoP* or ∆*phoQ* strains when inoculated in LB broth or minimal E salts medium supplemented with malic acid (Fig. 1C and Fig. S1), which is consistent with the notion that the PhoP response regulator boosts the antinitrosative potential of *Salmonella* in conjunction with its cognate PhoQ sensor kinase. The growth of ∆*phoQ Salmonella* in E salts was completely inhibited by 250 µM sperNO, which corresponded to the dose of sperNO that resulted in significant lethality when cells were challenged in PBS (Fig. 1 and Fig. S1). The hypersensitivity of ∆*phoQ Salmonella* to sperNO does not appear to be due defects in viability, as wild-type and ∆*phoQ Salmonella* strains grew with similar kinetics in LB and various minimal E salts media in the absence of sperNO (Fig. S2). Complementation of ∆*phoQ Salmonella* with a plasmid encoding a wild-type allele of *phoQ* (pPhoQ) restored wild-type levels of growth following sperNO treatment (Fig. 1C). Collectively, these data indicate that the PhoPQ two-component regulatory system contributes to the protection of *Salmonella* against the cytotoxic activity associated with RNS.

### Oxygen is required for the NO-dependent killing of *phoQ*-deficient *Salmonella*

**Figure 2:**
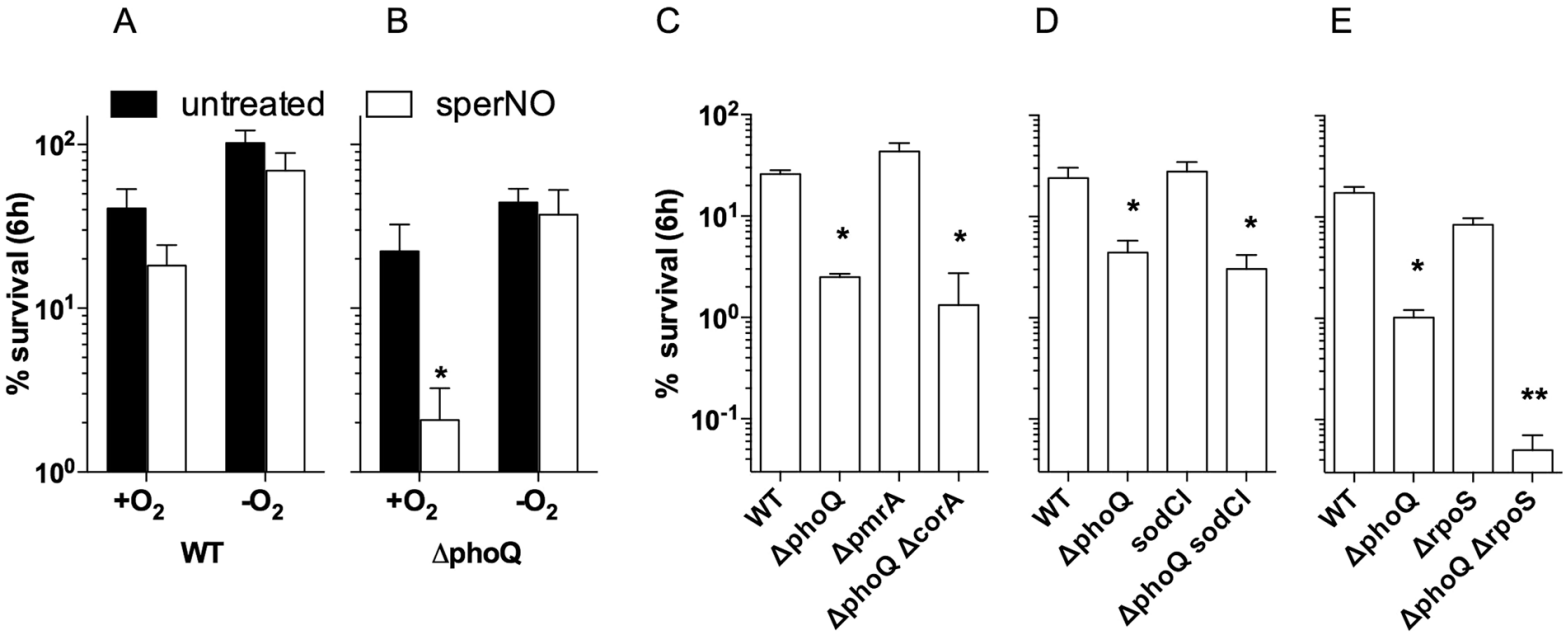
Oxygen is required for the NO-dependent killing of *phoQ*-deficient *Salmonella*. The survival of wild-type (WT) and ∆*phoQ Salmonella* strains after incubation at 37 °C for 6 h in the presence or absence of 250 µM sperNO was compared under normoxic (+O_2_) and low oxygen (−O_2_) conditions (**A**,**B**). The contribution of the PmrA response regulator, the CorA metal transporter, the copper-zinc superoxide dismutase (SodCI) and the alternative sigma factor RpoS to the resistance of *Salmonella* to 250 µM sperNO is shown in (**C**,**D** and **E**), respectively. *P < 0.05 compared to WT. **P < 0.05 compared to the ∆*phoQ* strain.

RNS including nitrogen dioxide (NO_2_^•^), N_2_O_3_ and ONOO^−^ produced in the reaction of NO with O_2_ and O_2_^•−^ indirectly mediate NO cytotoxicity^23^. To determine whether killing of ∆*phoQ Salmonella* by sperNO is mediated by NO itself or by a variety of RNS, *Salmonella* were exposed to 250 µM sperNO in the presence or absence of O_2_. To generate a hypoxic environment, PBS was flushed with N_2_ for 10 min and the experiments were carried out in sealed tubes. The viability of wild-type *Salmonella* was not (P > 0.05) affected by sperNO in either normoxic or hypoxic conditions (Fig. 2A). In contrast, the NO-dependent killing of ∆*phoQ Salmonella* was completely abrogated in hypoxic cultures (Fig. 2B). These findings suggest that the PhoPQ two-component regulatory system protects *Salmonella* against nitrooxidative products formed in the reaction of NO and O_2_ metabolites.

The antioxidant defenses associated with PhoPQ signaling rely on the expression of a functional PmrAB two-component regulatory system and the CorA metal transporter^7,24^. The susceptibility of ∆*phoQ Salmonella* to NO appears, however, to be independent of *pmrA* (Fig. 2C), which is consistent with previous investigations that reported that a *pmrA* mutant is as resistant to sperNO as wild-type controls^24^. The hypersusceptibility of *phoP* mutants to Fe^2+^-mediated oxidative stress can be prevented by a mutation in the *corA* metal transporter^7^. However, a mutation in *corA* did not prevent the killing of ∆*phoQ Salmonella* by sperNO (Fig. 2C). In addition to contributing to iron homeostasis, PhoPQ can activate *Salmonella*’s antioxidant defenses through the positive regulation of SodCI expression and the stabilization of RpoS^11,12^. However, neither SodCI or RpoS appear to contribute to the increased susceptibility of ∆*phoQ Salmonella* under the experimental conditions tested here (Fig. 2D and E). Interestingly, a strain of *Salmonella* lacking both *phoQ* and *rpoS* was even more susceptible to the RNS-dependent cytotoxicity than the *phoQ* mutant, suggesting that in the absence of PhoPQ the alternative sigma factor RpoS assumes a critical role in the regulation of the antinitrosative defenses of *Salmonella*.

### *Salmonella* exposed to NO undergoes nitrooxidative stress

**Figure 3:**
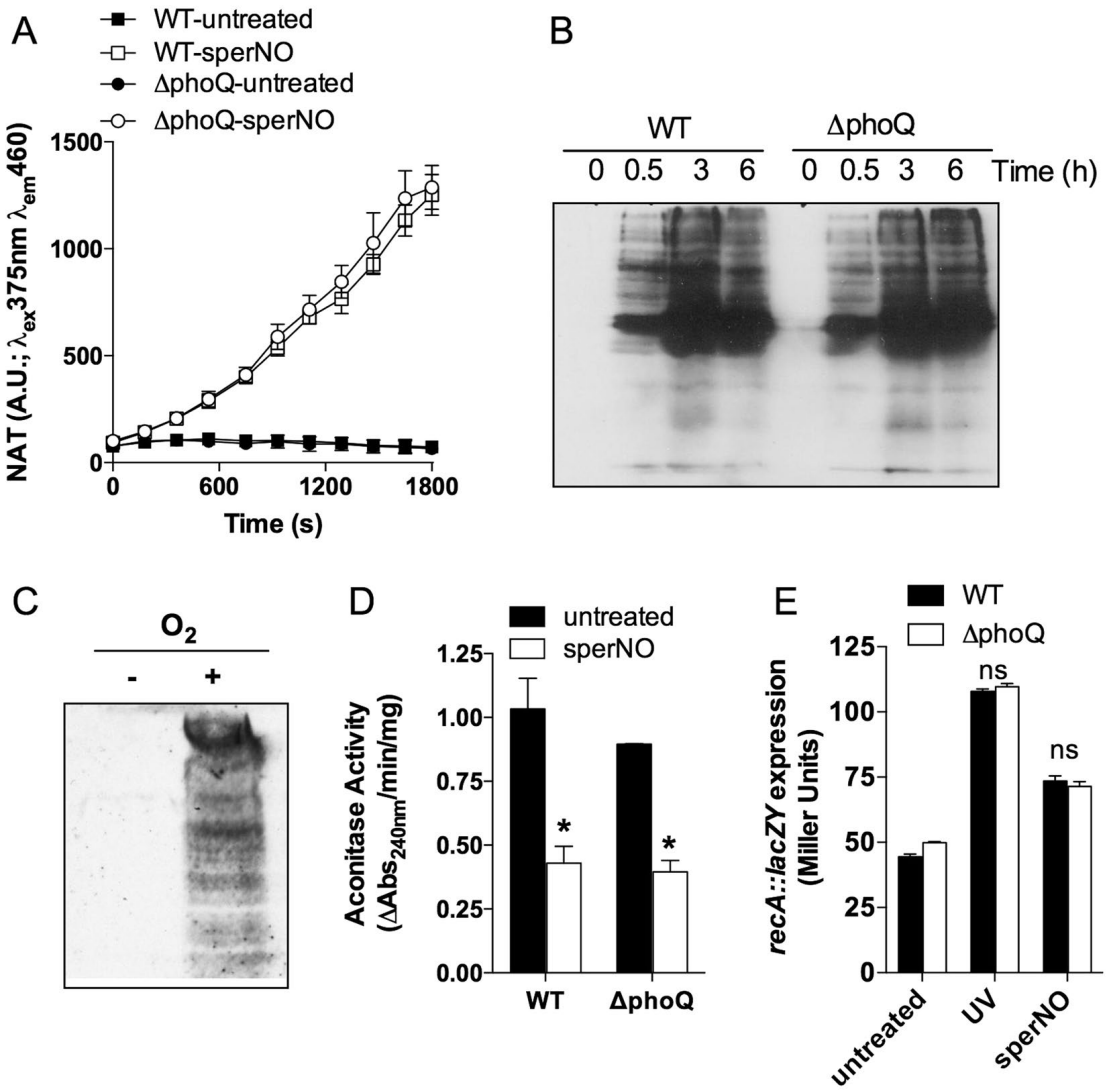
Generation of RNS in NO-treated *Salmonella*. N_2_O_3_ was quantified by following the generation of naphthyltriazol (NAT) in 5 × 10^5^ CFU ml^−1^ of wild-type (WT) and ∆*phoQ Salmonella* strains cultured in PBS at 37 °C in the presence or absence of 250 µM sperNO (**A**). The results are expressed as the mean arbitrary units (A.U.) ± S.D. representing 3 biological replicates from 2 independent experiments. Nitrotyrosine formation in whole-cell lysates isolated from ~ 2 × 10^8^ CFU ml^−1^ of WT and ∆*phoQ Salmonella* strains exposed to 250 µM sperNO in PBS at 37 °C was measured by Western blotting (**B**). A cropped image of a western blot showing the effect of O_2_ on the generation of nitrotyrosine in WT *Salmonella* exposed to 250 µM sperNO for 6 h is shown in (**C**). The NO-dependent damage of [Fe-S] cluster-containing dehydratases was monitored by following aconitase activity from cell lysates harvested from WT and *phoQ-*deficient *Salmonella* strains after exposure to 250 µM sperNO for 6 h at 37 °C in PBS (**D**). The expression of a *recA::lacZY* transcriptional fusion was quantified using β-galactosidase activity assays in wild-type (WT) and ∆*phoQ Salmonella* strains following exposure to 12 J/m^2^ ultraviolet light (UV) for 30 s or to 2.5 mM sperNO (**E**). Data in D & E represent the mean ± S.D. of 3 biological replicates. Statistical analysis was performed using a two-way ANOVA for data. *P < 0.001 compared to untreated controls; ns, no statistically significant difference compared to untreated controls.

To determine whether wild-type and ∆*phoQ Salmonella* experience different degrees of nitrooxidative stress upon exposure to sperNO, we monitored the formation of N_2_O_3_ (a reactive species generated upon autooxidation of NO in the presence of O_2_) and nitrotyrosine (an oxidative signature of the reaction of ONOO^−^ or other RNS with tyrosyl residues). Similar concentrations of N_2_O_3_ were generated after treatment of wild-type or ∆*phoQ Salmonella* with 250 µM sperNO (Fig. 3A). Substantial nitrotyrosine formation was also detected within 30 min after *Salmonella* were challenged with sperNO (Fig. 3B). Moreover, the profiles and kinetics of nitrotyrosine formation were similar in both wild-type and ∆*phoQ Salmonella* strains (Fig. 3B). Nitrotyrosine formation was not observed in low O_2_ cultures (Fig. 3C), suggesting that ONOO^−^ arising from the diffusion-limited reaction of exogenous NO with endogenous O_2_^•−^ is a likely candidate for the covalent oxidation of tyrosine residues in our experiments. In addition to tyrosine residues, [4Fe-4S] clusters of dehydratases are among the most avid targets (k = 1.4 × 10^5^ M^−1^ s^−1^) of ONOO^−^^25^. We therefore monitored the enzymatic activity of the [4Fe-4S] cluster-containing aconitase as a surrogate marker of ONOO^−^ mediated oxidative stress. Wild-type and ∆*phoQ Salmonella* harbored comparable basal levels of aconitase activity (Fig. 3D). Moreover, the aconitase activity of both wild-type and ∆*phoQ Salmonella* was similarly inhibited 6 h after exposure to sperNO (Fig. 3D). Together, these findings indicate that 250 µM sperNO exert substantial nitrooxidative stress on *Salmonella*. However, wild-type and ∆*phoQ Salmonella* appear to be exposed to similar levels of nitrooxidative species under the experimental conditions tested here. This idea is further substantiated by the degree of NO-dependent genotoxicity seen in these two strains of *Salmonella*. DNA damage was indirectly measured by following the expression of a transcriptional *lacZY* fusion to the SOS response *recA* gene. The *recA::lacZY* transcriptional fusion was similarly induced in both wild-type and ∆*phoQ Salmonella* after exposure to 12 J/m^2^ UV light or treatment with 2.5 mM sperNO (Fig. 3E).

### Mutations in NADH dehydrogenases NDH-I and NDH-II protect ∆*phoQ Salmonella* from NO-dependent cytotoxicity and protein nitration

**Figure 4:**
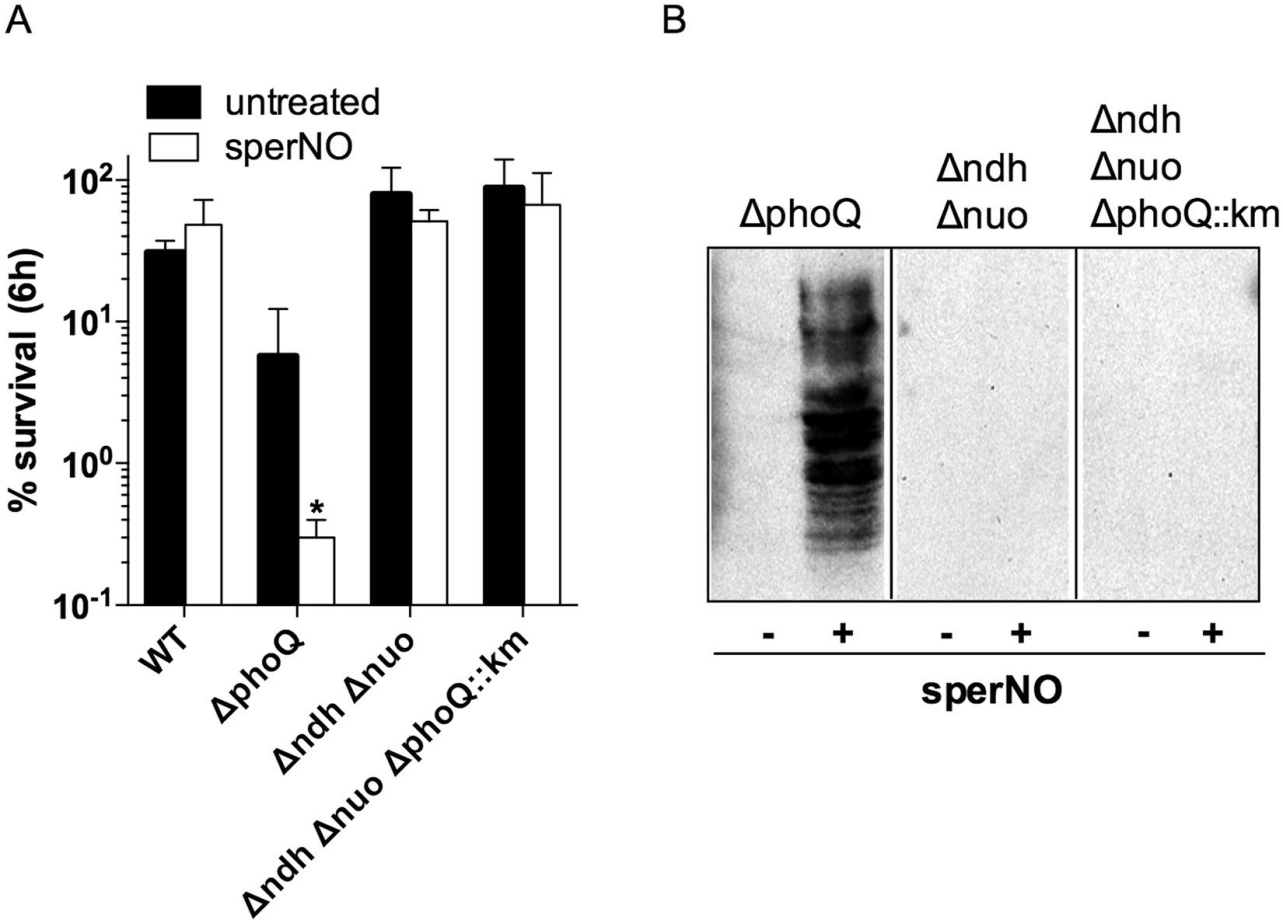
Defects in NADH dehydrogenases protect *phoQ*-deficient *Salmonella* from NO-dependent cytotoxicity. The survival of WT, Δ*phoQ*, Δ*ndh* Δ*nuo*, and Δ*ndh* Δ*nuo* Δ*phoQ*::*km* strains after incubation with 250 µM sperNO in PBS at 37 °C for 6 h (**A**). The data in A are represented by the mean % survival ± S.D. of 6 independent observations from 2 separate experiments. Statistical analysis was performed using a two-way ANOVA for data. *P < 0.001 compared to untreated controls. The presence of nitrotyrosine in whole-cell lysates of the indicated *Salmonella* strains incubated in the presence or absence of 250 µM sperNO in PBS at 37 °C for 6 h was determined by Western blot (**B**). The data presented in B are presented as cropped panels for each individual strain tested, and are representative of two independent experiments.

We examined in more detail the mechanism underlying the cytotoxicity of RNS against ∆*phoQ Salmonella*. NADH dehydrogenases of the electron transport chain can be a sizable source of oxidative stress in the cell^26^. We tested whether the sperNO-mediated, O_2_-dependent killing of ∆*phoQ Salmonella* was the result of the synergism between exogenous NO and O_2_^•−^ arising from the adventitious reduction of O_2_ by NADH dehydrogenases of the electron transport chain. To test this hypothesis, the *∆phoQ::km* mutant allele was introduced into the *∆nuo ∆ndh* mutant strain AV0438 lacking both NDH-I and NDH-II NADH dehydrogenases. As noted for H_2_O_2_ and ONOO^−^^27^, the complex I-deficient *∆nuo ∆ndh* strain AV0438 was resistant to 250 µM sperNO (Fig. 4A). Strikingly, strain AV0810 harboring mutations in *phoQ, nuo* and *ndh* was also resistant to NO (Fig. 4A). Similar to the complex I-deficient isogenic strain AV0438, strain AV0810 lacking *phoQ, nuo* and *ndh* appear to be protected from ONOO^−^ as indicated by a lack of nitrotyrosine formation 6 h after exposure to 250 µM sperNO (Fig. 4B). Collectively, these data indicate that ONOO^−^ dependent nitrooxidative stress engendered upon reaction of exogenous NO with O_2_^•−^ produced by NADH dehydrogenases of the electron transport chain contributes to the NO-mediated killing of ∆*phoQ Salmonella*.

### Exogenous Mg^2+^ rescues ∆*phoQ Salmonella* from RNS-dependent killing

**Figure 5:**
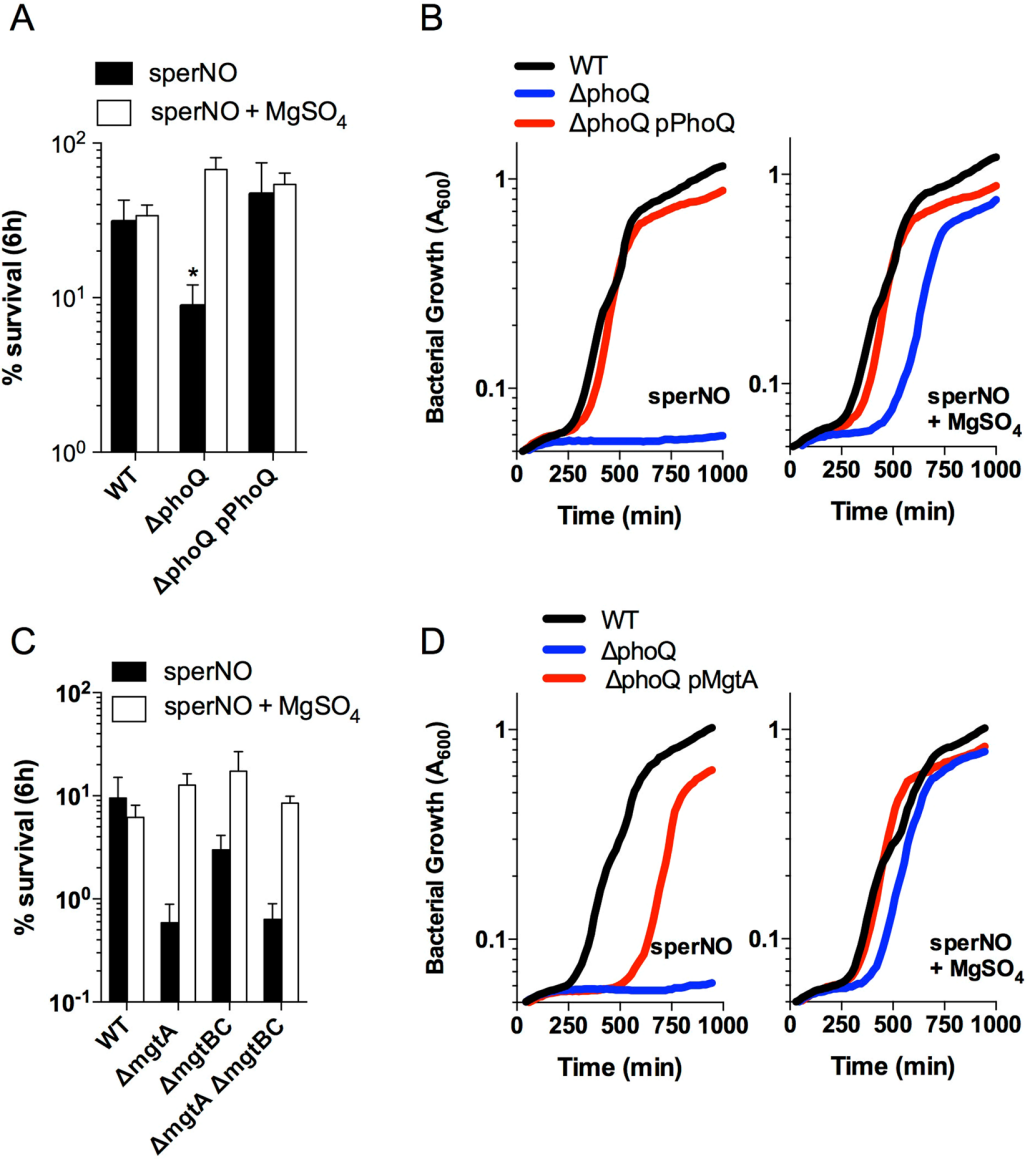
PhoPQ-dependent regulation of Mg^2+^ homeostasis protects *Salmonella* from RNS cytotoxicity. The survival of WT, Δ*phoQ*, and Δ*phoQ* pWSK29::*phoQ* (pPhoQ) *Salmonella* strains was determined after incubation of strains with 250 µM sperNO in the presence or absence of 10 mM MgSO_4_ in PBS at 37 °C for 6 h (**A**). The data, which are represented as mean ± S.D., are from 8 replicates collected from 2 separate experiments. *P < 0.01 compared to sperNO-treated WT controls. The OD_600nm_ was measured over time as described in Fig. 1 to determine the growth of *Salmonella* strains cultured in LB + 2.5 mM sperNO, or LB + 2.5 mM sperNO +10 mM MgSO_4_ at 37 °C. The survival of WT, Δ*mgtA::km*, Δ*mgtBC*, and Δ*mgtBC* Δ*mgtA::km Salmonella* strains was determined following incubation with 250 µM sperNO in the presence or absence of 10 mM MgSO_4_ in PBS at 37 °C for 6 h (**C**). The data are represented as mean % survival ± SD from 8 replicates collected from 2 separate experiments. *P < 0.01 compared to sperNO-treated WT controls. The OD_600nm_ was measured over time as described in Fig. 1 to determine the growth of *Salmonella* strains cultured in LB + 2.5 mM sperNO or LB + 2.5 mM sperNO + 10 mM MgSO_4_ at 37 °C (**D**). Data represent the mean OD_600nm_ of 3 biological replicates.

Previously, *Salmonella* was shown to exhibit increased susceptibility to oxidative stress following disruptions in Mg^2+^ uptake through mutations of *phoP* or the PhoPQ-regulated Mg^2+^ transporters *mgtA* and *mgtB* ^7^. Therefore, we investigated whether the increased susceptibility of ∆*phoQ Salmonella* to RNS was due to disruptions in Mg^2+^ homeostasis. The increased susceptibility of the ∆*phoQ Salmonella* strain to killing by 250 µM sperNO in PBS was prevented by the addition of 10 mM MgSO_4_, but had no effect on the survival of the wild-type strain or the *∆phoQ* strain complemented with a pPhoQ plasmid (Fig. 5A). Moreover, the sperNO-dependent inhibition of growth of ∆*phoQ Salmonella* was alleviated by the addition of 10 mM MgSO_4_ when cultured in LB with 2.5 mM sperNO (Fig. 5B). This protective effect of exogenous MgSO_4_ was also observed in *∆phoQ Salmonella* challenged with 250 µM sperNO in minimal E salts media supplemented with glucose, malic acid, or fumarate (Fig. S2). Moreover, the addition of MgCl_2_ also restored the growth of *∆phoQ Salmonella* challenged with sperNO, while the addition of CaCl_2_ had no effect (Fig. S3). Collectively, these data suggest that the hypersensitivity of ∆*phoQ Salmonella* to RNS is due to disruptions in Mg^2+^ homeostasis.

We hypothesized that the increased susceptibility of the *∆phoQ Salmonella* strain to RNS was linked to its inability to upregulate the expression of Mg^2+^ transporters encoded by *mgtA* and *mgtB*. Therefore, we compared the susceptibility of *Salmonella* strains lacking *mgtA* and/or *mgtBC* to killing by 250 µM sperNO. The ∆*mgtA*-deficient *Salmonella* strain showed a significantly increased susceptibility to killing by sperNO compared to both wild-type and *∆mgtBC* strains (Fig. 5C). A strain harboring mutations in both *mgtA* and *mgtBC* was no more susceptible to killing by RNS as the ∆*mgtA* mutant strain suggesting that the PhoPQ-dependent regulation of *mgtA* promotes resistance to RNS. As was the case for the *∆phoQ Salmonella* strain (Fig. 5A), the addition of 10 mM MgSO_4_ prevented sperNO-dependent killing of the ∆*mgtA Salmonella* (Fig. 5D). These data suggested that the increased susceptibility of ∆*phoQ Salmonella* to RNS was due to its inability to upregulate the expression of *mgtA*, which is required for proper Mg^2+^ homeostasis. To test this hypothesis, we introduced a pBAD/HisA plasmid encoding *mgtA* (pBmgtA) under an arabinose-inducible promoter into the *∆phoQ Salmonella* strain, and compared its ability to grow in LB broth in the presence or absence of sperNO. The wild-type, *∆phoQ*, and *∆phoQ* pMgtA strains showed similar growth kinetics in LB broth (Fig. 5D). As described earlier, Δ*phoQ Salmonella* were unable to grow in LB broth in the presence of 2.5 mM sperNO (Figs 1C and 5D). In contrast, the introduction of a pMgtA plasmid to the *∆phoQ* strain restored growth in LB broth in the presence of 2.5 mM sperNO, albeit with an increased lag compared to the wild-type control (Fig. 5D). This lag was eliminated by the addition of 10 mM MgSO_4_, suggesting that the expression of *mgtA* from the pMgtA plasmid could only partially restore Mg^2+^ homeostasis in the *∆phoQ* strain. Collectively, these data support the hypothesis that PhoPQ promotes resistance to nitrooxidative stress in *Salmonella* through the regulation of Mg^2+^ homeostasis.

## Discussion

The PhoP regulon controls the antioxidant defenses of *Salmonella*, *Yersinia pestis* and *Enterococcus faecalis* ^12,28,29^, and work from our laboratory indicates that this two-component regulatory system also is contributes to the antinitrosative defenses of *Salmonella* ^13^. Elegant investigations by Dr. Groisman’s group have elucidated that the PhoP regulon defends *Salmonella* against oxidative stress engendered in the reduction of H_2_O_2_ by the Fenton catalyst Fe^2+7^. Little is known, however, about the nitrosative chemistry antagonized by this two-component regulatory system. We therefore deemed it important to investigate the newly described function of PhoPQ in the antinitrosative defenses of *Salmonella*. The investigations presented here are consistent with a model in which the PhoPQ two-component regulatory system antagonizes the antimicrobial activity of ONOO^−^. In support of this model, the sperNO-mediated killing of ∆*phoQ Salmonella* is restricted to aerobic cultures, and coincides with the formation of nitrotyrosine, and the inactivation of the TCA cycle enzyme aconitase. These observations can be explained if one takes into account that the generation of ONOO^−^ requires the reaction of O_2_^•−^ and NO. O_2_^•−^ is formed adventitiously at the flavin or quinone-binding sites of NADH dehydrogenases of the electron transport chain and its production requires O_2_. The ONOO^−^ produced in the reaction of endogenous O_2_^•−^ and exogenous NO is a powerful nitrating and oxidizing agent that could explain the formation of nitrotyrosine residues in cytoplasmic proteins and the oxidation of the [4Fe-4S] clusters of dehydratases. The protection afforded by mutations in NADH dehydrogenases against ONOO^–^ dependent cytotoxicity could be explained by three independent and complementary mechanisms. First, the accumulation of NADH in ∆*ndh* ∆*nuo Salmonella* effectively scavenges NO_2_^•^ and OH^•^ radicals caged in peroxynitrous acid (ONO-OH), which is the dominant ONOO^−^ congener at the neutral pH of the bacterial cytoplasm^27^. Second, NADH fuels the enzymatic detoxification of ONOO^−^ by the AhpCF alkylhydroperoxidase^27,30^. And third, a lack of NADH dehydrogenases diminishes ONOO^−^ synthesis by limiting the flow of electrons through the respiratory chain that is required for the generation of O_2_^•−^. Our investigations suggest that ONOO^−^ is necessary but not sufficient for the NO-mediated antimicrobial activity, because wild-type *Salmonella* and the *phoQ* mutant bacteria suffer a similar degree of nitrotyrosine formation and inactivation of aconitase upon exposure to sperNO, but are differently killed by the oxidative congeners of this diatomic radical.

[4Fe-4S] clusters of dehydratases can be directly nitrosylated by NO at a rate constant of 10^6^ M^−2^ sec^−1^ ^31^. Because the nitrosylation of [4Fe-4S] clusters is second order for NO, this chemistry is less likely to occur at the low NO fluxes sustained in the course of our investigations. The inactivation of [4Fe-4S] clusters by ONOO^−^, which occurs at the fast rate of 1.4 × 10^5^ M^−1^ sec^−1^ ^25^, is first order for ONOO^−^. The speed of the reaction indicates that the oxidation of [4Fe-4S] clusters by ONOO^−^ is limited by the production of this RNS. NO and O_2_^•−^ react with a second order rate constant of 10^9^ M^−1^ sec^−1^ to form ONOO^−^^32^. However, high concentrations of NO readily consume ONOO^−^. Therefore, generation of ONOO^−^ in the *Salmonella* cytoplasm is most likely to be maximal at the low rates of NO synthesis supported during the innate immune response. In the presence of a functional PhoPQ two-component regulatory system the ONOO^−^ produced endogenously appears to be tolerated by *Salmonella*.

The Hmp-mediated and cytochrome *bd*-mediated detoxification of NO, the stringent response, low-molecular weight thiols, along with DNA repair systems minimize the cytotoxicity of NO produced in the innate response to *Salmonella* ^23,33–35^. Our investigations identify PhoPQ-dependent regulation of Mg^2+^ homeostasis as an additional antinitrosative defense that shields *Salmonella* from the cytotoxicity of low NO fluxes. While the hypersensitivity of ∆*phoQ Salmonella* to RNS is tied to disrupted Mg^2+^ homeostasis, this phenotype appears to be independent of both PmrAB-dependent and CorA-dependent resistance Fe^2+^ toxicity. This conclusion is supported by the fact that 1) *pmrA* or *corA* single mutant strains do not exhibit increased sensitivity to RNS compared to wild-type strains, and 2) *phoQ pmrA* or *phoQ corA* double mutant *Salmonella* strains were as sensitive to killing by sperNO as ∆*phoQ Salmonella*. Cellular concentrations of Mg^2+^ in *Salmonella* are capable of reaching 100 mM^36^, with the majority of Mg^2+^ bound to ribosomes and nucleotide triphosphates^37^. Therefore, the reduced cytoplasmic Mg^2+^ concentrations in ∆*phoQ Salmonella* may increase the susceptibility to killing by RNS due to several factors including 1) reduced protein synthesis resulting from the dissociation of Mg^2+^ from ribosomes, and 2) leaching of Mg^2+^ from nucleotide triphosphates preventing their use as substrates for enzymatic reactions necessary to repair RNS-induced cellular damage. This is supported by the fact that the addition of 10 mM MgSO_4_ rescued both ∆*phoQ* and ∆*mgtA Salmonella* strains from RNS-dependent killing.

Independent of the regulation of antioxidant defenses or targets of nitrooxidative stress, a functional PhoPQ two-component regulatory system is likely to promote antinitrosative defenses through the activation of SPI2 transcription^38–40^, because the SPI2 type III secretion system has been shown to minimize fusion of *Salmonella*-containing vacuoles with vesicles harboring iNOS^41^. In contrast to the concerted and rich repertoire of antinitrosative defenses that protect *Salmonella* against the low NO fluxes produced in the innate response, no antinitrosative defenses are known to protect this facultative intracellular pathogen against the massive nitrosative stress unleashed in IFNγ-activated macrophages. Paradoxically, N_2_O_3_ and other high oxidation NO congeners generated by IFNγ-primed macrophages exert profound anti-*Salmonella* activity by repressing SPI2 transcription and PhoPQ signaling^13,16,17^. In turn, RNS-dependent repression of PhoPQ signaling and SPI2 transcription promotes the maturation of the *Salmonella* phagosome along the degradative pathway for fusion with lysosomes.

**Figure 6:**
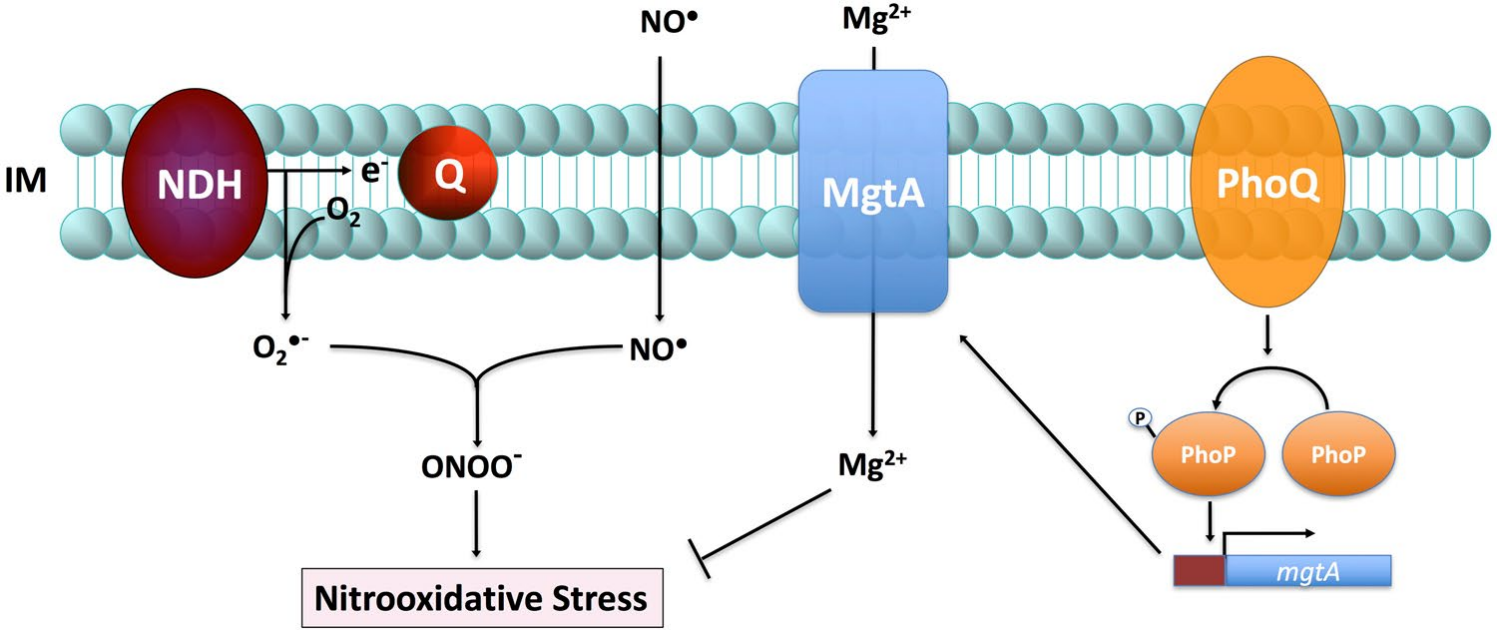
Model for PhoPQ-mediated resistance to nitrooxidative stress. Our investigations herein and elsewhere indicate that the PhoPQ signaling cascade protects *Salmonella* against the antimicrobial activity of nitric oxide (NO) produced by the inducible NO synthase (iNOS). NADH dehydrogenases (NDH) at the inner membrane (IM) transfer electrons (e^−^) from NADH to the quinone pool in the electron transport chain. Although this process is remarkably efficient, some electrons adventitiously reduce molecular oxygen (O_2_) to generate the superoxide radical (O_2_^•−^). The diffusion limited reaction of O_2_^•−^ and exogenous NO. gives rise to peroxynitrite (ONOO^−^) generating nitrooxidative stress. The PhoPQ two-component regulatory system protects *Salmonella* against nitrooxidative stress by maintaining optimal cytoplasmic concentrations of Mg^2+^ through the regulation of the Mg^2+^ transporter MgtA.

In summary, this study has revealed that in addition to its known roles in protecting *Salmonella* from acid pH, bile salts, antimicrobial peptides, and oxidative stress^1,4–10^, the PhoPQ two-component regulatory system contributes to the resistance of *Salmonella* against the nitrooxidative stress generated in the reaction of exogenous NO and endogenously produced O_2_^•−^ by maintaining Mg^2+^ homeostasis (Fig. 6).

## Methods

### Bacterial Strains

*Salmonella enterica* serovar Typhimurium strain ATCC 14028 s was used throughout this study as wild-type, and as a background for the construction of mutations and a *recA::lacZY* transcriptional fusion (Table 1). The mutations were generated following the one-step, λ-Red-mediated gene replacement method of Datsenko and Wanner^42^. Briefly, primers encoding 40–42 nucleotides homologous to the target gene followed by 20 nucleotides homologous to the pKD13 template plasmid were used for the PCR amplification of the Flp recombinant target (FRT)-flanked kanamycin resistance cassette. The resulting PCR products were DpnI digested and electroporated into *S*. Typhimurium strain TT22236 carrying the pTP2223 plasmid that expresses the λ-Red recombinase under Ptac control. Mutations were moved between strains by P22-mediated transduction and pseudolysogens eliminated by streaking on Evans blue uranine agar plates. Nonpolar deletions were generated by recombining the two FRT sites flanking the kanamycin resistance cassette with the Flp recombinase encoded by the pCP20 plasmid^43^. The mutations were confirmed by PCR analysis. A *recA::lacZY* transcriptional fusion was constructed by the pCP20-mediated integration of pCE36 encoding a promoterless *lacZY* operon into the unique FRT scar engineered immediately downstream of the *recA* stop codon. The pMgtA plasmid was generated by cloning a wild-type copy of the magnesium transporter *mgtA* into the pBAD/HisA vector using the primers listed in Table 2. The pMgtA plasmid was electroporated into *Salmonella* strain AV0475 carrying a ∆*phoQ::FRT* allele to produce the TJB1301 strain.

**Table 1:** Bacterial Strains and Plasmids.

Strains	Description	Reference
*Salmonella enterica* serovar Typhimurium strain 14028 s	Wild-type	ATCC
TT22236	LT2 *Salmonella* carrying pTP2223	(Price-Carter *et al*., 2001)
AV0436	*∆ndh::FRT Δnuo::km*	(Husain *et al*., 2008)
AV0438	*∆ndh::FRT Δnuo::FRT*	This study
AV0474	∆*phoP::FRT*	(Bourret *et al*., 2008)
AV0462	∆*phoQ::km*	This study
AV0475	∆*phoQ::FRT*	(Bourret *et al*., 2008)
AV0560	∆*phoQ::FRT* pPhoQ	(Bourret *et al*., 2008)
AV06108	∆*rpoS::km*	(Bourret *et al*., 2008)
TJB0601	∆*phoQ::FRT* ∆*rpoS::km*	This study
AV07174	∆*corA::km*	This study
AV07175	∆*phoQ::FRT* ∆*corA::km*	This study
AV07234	*recA-ter::FRT* pCP20	This study
AV07235	*recA::lacZY*	This study
AV07250	∆*phoQ::FRT recA::lacZY*	This study
AV0810	∆*ndh::FRT Δnuo::FRT ΔphoQ::km*	This study
MF1005	*sodCI::Tn10*	(De Groote *et al*., 1997)
TJB0602	∆*phoQ::FRT sodCI::Tn10*	This study
AV13037	∆*mgtA::km*	This study
AV13035	∆*mgtBC::km*	This study
AV13036	∆*mgtBC::FRT*	This study
AV13038	∆*mgtBC*::*FRT* ∆*mgtA::km*	This study
TJB1301	∆*phoQ::FRT* pMgtA	This study
**Plasmids**		
pTP2223	Plac *lam bet exo* tet^R^	(Poteete and Fenton, 1984)
pCP20	*bla cat cI*857 P_R_ *flp* pSC101 oriTS	(Cherepanov and Wackernagel, 1995)
pKD13	*bla* FRT *ahp* FRT PS1 PS4 oriR6K	(Datsenko and Wanner, 2000)
pCE36	*ahp* FRT *lacZY* ^+^ t_*his*_ oriR6K	(Ellermeier *et al*., 2002)
pWSK29	*bla lacZ* oripSC101	(Wang *et. al*., 1991)
pWSK29::*phoQ* (pPhoQ)	*bla lacZ* oripSC101	(Bourret *et al*., 2008)
pBAD/HisA	*bla* oripBR322 *araC*	Thermo Fisher
pBAD/HisA::*mgtA* (pMgtA)	*bla* oripBR322 *araC*	This study

**Table 2:** Primers.

Name	Sequence
*rpoS*-pKD13-F	F:5′-CATGATTTAAATGAAGACGCGGAATTTGATGAGAACGGAGCTGGAGCTGCTTCGAAGTT
*rpoS-*pKD13-R	R:5′-GCCTTCAACCTGAATCTGACGAACACGTTCACGCGTAAGTTCCGGGGATCCGTCGACCT
*corA*-pKD13-F	F:5′-TGAACTGTCCGATATTTTTACGCATTGGGAGTCCCGGTCAGCTGGAGCTGCTTCGAAGTT
*corA*-pKD13-R	R:5′-TTCAGCCGCAGCTGAATCACCCTGGCCTTAATGTCTTATTCCGGGGATCCGTCGACCT
*mgtA*-pKD13-F	F:5′-TCTGCGCCTGACTTCGGCGCGGAGGGATTACCTATGCTAGTGTAGGCTGGAGCTGCTTC
*mgtA*-pKD13-R	R:5′-AGCACGCTGGCGAATCCCCGACGAAAGTGTTTACTGCCAATTCCGGGGATCCGTCGACC
*mgtBC*-pKD13-F	F:5′-GTGTGCTAAATATAGCACGTACTTATTCTTCCAGAAAAAATGGAGGTGTAGGCTGGAGCTGCTTC
*mgtBC*-pKD13-R	R:5′-TGAGCGATTCATCTGGGCGATCCTCAAACATTATTAAAACCAATTCCGGGGATCCGTCGACC
*recA-*pKD13-F	F:5′-GACGATAGCGAAGGCGTTGCAGAAACCAACGAAGATTTTTAATGGCTGGAGCTGCTTCGAAGTT
*recA-*pKD13-R	R:5′-ATGGCGGCTTCGTTTTGCCCGCCCCACCATCACCTGATGATTCCGGGGATCCGTCGACCT
*mgtA*-XhoI-F	F:5′-ACGTAGCTCGAGCTCGAGCTAAAAATCATTACCCGC
*mgtA*-EcoRI-R	R:5′-ACGTAGGAATTCTTACTGCCAGCCATAACGTCT

### Susceptibility of *Salmonella* to reactive nitrogen species

*Salmonella* strains were inoculated from frozen stocks and grown in Luria-Bertani (LB) broth with shaking at 325 r.p.m. at 37 °C for 20 h. Strains were then diluted in PBS to a concentration of ~5 × 10^5^ cells ml^−1^. The bacteria were challenged at 37 °C with 250 μM of the NO donor spermine NONOate (Cayman Chemical, Ann Arbor, MI). Selected groups of bacteria were challenged at 37 °C with 250 μM spermine NONOate in PBS that had been depleted of O_2_ after 10 min of flushing with N_2_. Percent survival was calculated by recording the number of bacteria capable of forming a CFU on LB agar plates. Alternatively, stationary phase cultures of the *Salmonella* strains were subcultured 1:200 in fresh LB broth in the presence or absence of 2.5 mM spermine NONOate. Bacterial suspensions were seeded in 96-well plates, and were grown at 37 °C with shaking at 282 r.p.m. with the optical density measured at 600 nm (OD_600nm_) using a Cytation 5 multi-mode plate reader (BioTek, Winooski, VT).

### Aconitase enzymatic assay

*Salmonella* strains grown in Luria-Bertani (LB) broth with shaking at 325 r.p.m. at 37 °C for 20 h were pelleted by centrifugation and resuspended in PBS to an OD_600nm_ of 0.5. Soluble cytoplasmic proteins were isolated from bacteria incubated at 37 °C for 6 h in PBS in the presence or absence of 250 μM spermine NONOate. Briefly, the bacteria were washed in 20 mM Tris-citrate buffer pH 8.0, and the cytoplasmic proteins extracted by sonication. Bacterial debris was removed by centrifugation at 14,000 RPM in a microcentrifuge for 30 s. Aconitase activity contained in the cytoplasmic extracts was estimated spectrophotometrically at 240 nm by following the formation of cis-aconitate in Tris-citrate buffer containing 20 mM isocitrate^44^. The protein concentration in the cytoplasmic extracts was measured by the BCA protein assay (Pierce, Rockford, IL). Aconitase activity is expressed as the mean OD_240nm_/min/mg protein ± SD of 2 independent experiments.

### Estimation of DNA damage

The accumulation of single strand and double strand DNA damage was indirectly estimated by measuring the expression of a *lacZY* transcriptional fusion of the SOS response *recA* gene. Selected groups of bacteria were irradiated with 12 J/m^2^ UV using a TL-2000 Ultraviolet Translinker (Ultraviolet Products, Upland, CA), or treated with 2.5 mM spermine NONOate at 37 °C for 6 h. Expression of the *recA::lacZY* transcriptional fusion was quantified spectrophotometrically as β-galactosidase enzymatic activity using the substrate o-nitrophenyl-β-D-galactopyranoside. β-galactosidase activity is expressed as Miller units using the equation 1,000 × [(OD_420nm_ − 1.75 × OD_550nm_)/(T_(min)_ × V_(ml)_ × OD_600nm_)].

### N_2_O_3_ quantification

The generation of N_2_O_3_ in *Salmonella* strains exposed to 250 μM spermine NONOate was determined indirectly by following the formation of the N-nitrosonapthalen derivative of 2,3-diaminonaphthalen (Sigma-Aldrich) as described^45^. A 100 mM stock of 2,3-diaminonaphthalen prepared in dimethylformamide was used at a final concentration of 200 μM in PBS. Accumulation of N-nitrosonapthalen was recorded for 30 min following treatment of *Salmonella* strains with spermine NONOate. Fluorescence was measured on a Synergy HT fluorometer (BioTek) set at λ_ex_ = 375 nm and λ_em_ = 460 nm.

### Detection of nitrotyrosine formation by Western blot analysis

*Salmonella* strains grown in Luria-Bertani (LB) broth with shaking at 325 r.p.m. at 37 °C for 20 h were pelleted by centrifugation, and resuspended in PBS to an OD_600_ of 0.5. Bacteria were incubated at 37 °C in the presence or absence of 250 μM spermine NONOate. At the specified timepoints, the bacteria were pelleted by centrifugation and resuspended in 200 μL of alkaline lysis buffer (25 mM Tris, 100 mM SDS, and 128 mM NaOH). The specimens were separated in 10% SDS-PAGE gels, transferred to nitrocellulose membranes, and probed with an anti-nitrotyrosine polyclonal antibody (Upstate, Lake Placid, NY) followed by a horseradish peroxidase-conjugated, anti-rabbit IgG secondary antibody. Detection was carried out using the Enhanced Chemiluminescence Kit (GE Healthcare, Piscataway, NJ) on a Molecular Imager Fx (BioRad, Hercules, CA).

### Statistical analysis

Data are presented as mean ± standard deviation (SD). To determine statistical significance between multiple comparisons, two-way analysis of variance (ANOVA) were performed, followed by a Bonferroni posttest. Data were considered statistically significant when *P* was < 0.05.

## Electronic supplementary material

Supplementary Material
