# Supplementary material for: Magnesium homeostasis protects Salmonella against nitrooxidative stress
Source: Sci Rep. 2017 Nov 8;7:15083. doi: 10.1038/s41598-017-15445-y (PMC5678156; doi:10.1038/s41598-017-15445-y)
Supplement: Supplementary file 1 — Supplementary Material [file 41598_2017_15445_MOESM1_ESM.pdf]

Magnesium homeostasis protects *Salmonella* against nitrooxidative stress

Travis J. Bourret[1*], Lin Liu[2], Jeff A. Shaw[1], Maroof Husain[2,3], & Andrés Vázquez-Torres[2,4]

[1]Department of Medical Microbiology and Immunology
2500 California Plaza
Creighton University
Criss I, Rm 521
Omaha, NE 68178

[2]Department of Immunology and Microbiology
University of Colorado School of Medicine
Aurora, CO, 80045

[3]Current address:
Division of Molecular and Translational Biomedicine
Department of Anesthesiology and Perioperative Medicine
University of Alabama at Birmingham
Birmingham, AL, 35249

[4] Veterans Affairs Eastern Colorado Health Care System
1055 Clermont Street
Denver, DO 80220, USA.

Supplementary Material

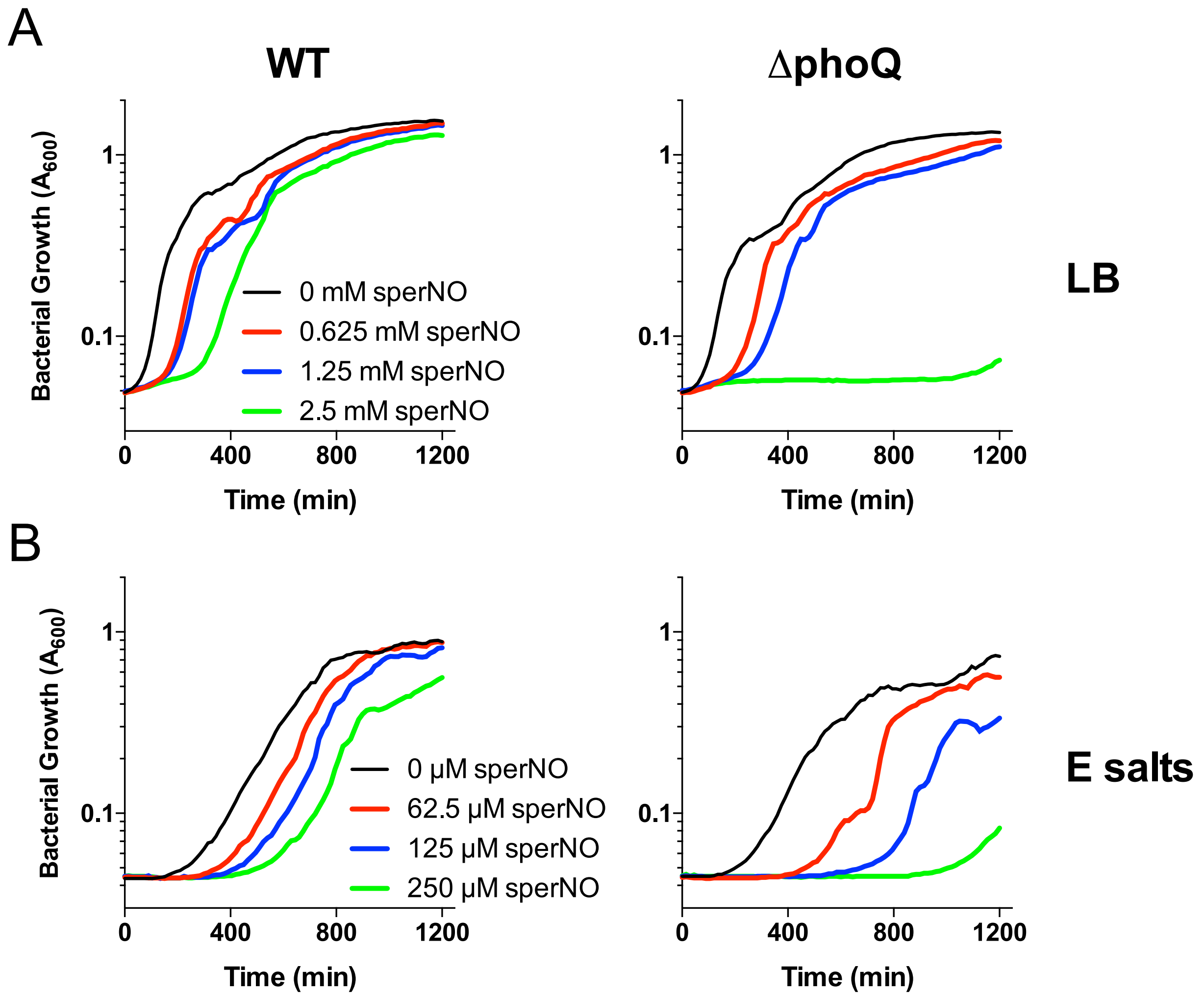

Figure S1. Effect of RNS on the growth of wild-type and *ΔphoQ Salmonella* in rich and minimal media. *Salmonella* strains grown for 20 h in LB at 37˚C with shaking at 325 r.p.m. were subcultured 1:200 in fresh LB **(A)** or minimal E salts (0.2 g/L $MgSO_4$, 2 g/L $C_6H_8O_7$-H_2O, 10 g/L K_2HPO_4, 3.5 g/L $Na(NH_4)HPO_4$-$4H_2O$, pH 7.0) + 5 mM malic acid **(B)** in the presence or absence of the indicated concentrations of spermine NONOate (sperNO). The data are presented as the mean bacterial growth from 3 biological replicates.

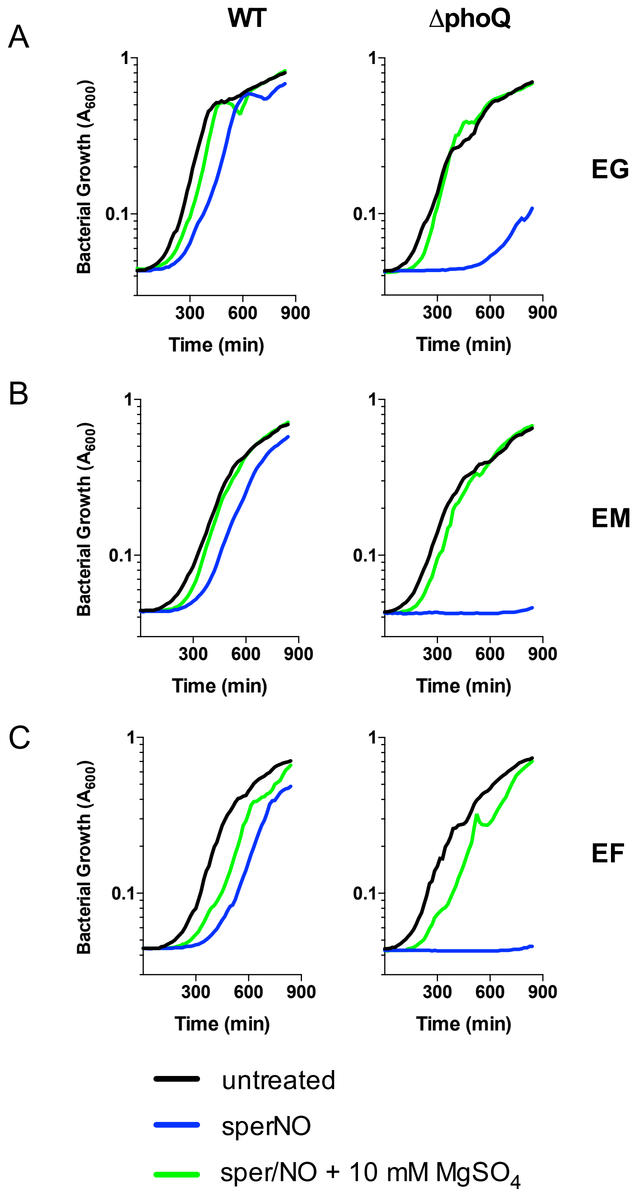

Figure S2. Growth inhibition of *ΔphoQ Salmonella* strains by RNS in minimal media is reversed by exogenous $MgSO_4$. Stationary phase *Salmonella* strains were cultured as described in Figure S1 and challenged with 250 µM spermine NONOate (sperNO) in minimal E salts supplemented with 5 mM glucose (EG) **(A)**, 5 mM malic acid (EM) **(B)**, or 5 mM fumarate (EF) **(C)**. Exogenous $MgSO_4$ was added to designated cultures at the time of challenge with sperNO. The data are presented as the mean bacterial growth from 3 biological replicates.

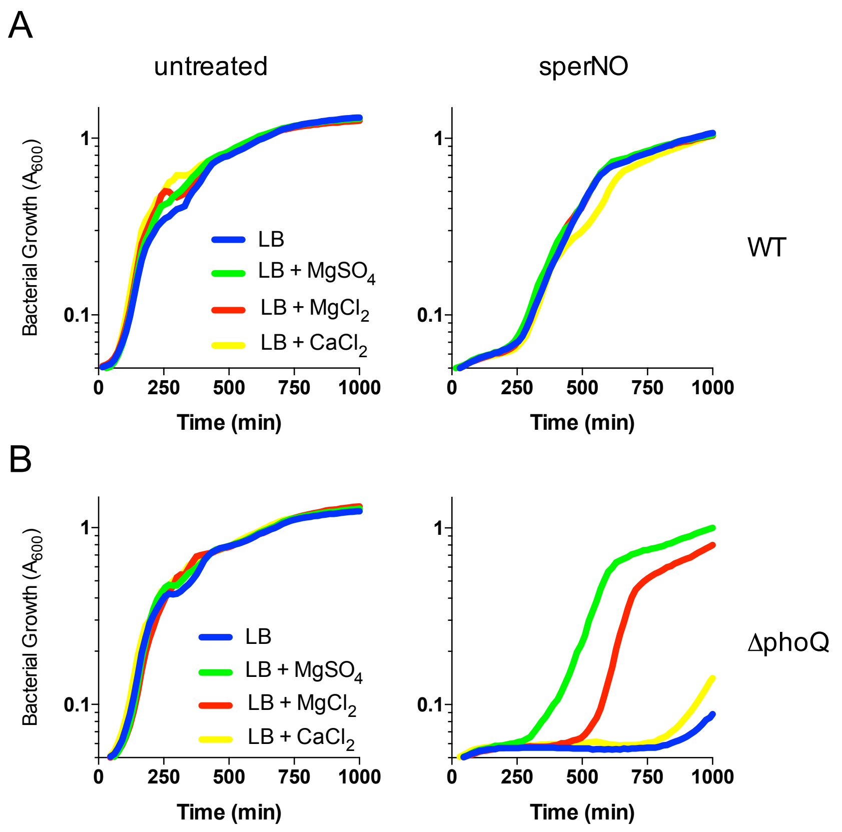

Figure S3. Effect of divalent cations on the growth of *Salmonella* strains following challenge with RNS. *Salmonella* strains were grown as described in Fig. S1, and grown in the presence or absence of 2.5 mM spermine NONOate (sperNO). Selected *Salmonella* cultures were supplemented with 10 mM $MgSO_4$, $MgCl_2$, or $CaCl_2$ at the time of challenge with sperNO. The data are presented as the mean bacterial growth from 3 biological replicates.
